# A Study of Airborne Pollen Grains and Fungal Spores in the Region of Epirus (Northwestern Greece)

**DOI:** 10.7759/cureus.26335

**Published:** 2022-06-26

**Authors:** Ioannis D Komnos, Maria C Michali, Nafsika V Ziavra, Michael A Katotomichelakis, Ioannis G Kastanioudakis

**Affiliations:** 1 Otorhinolaryngology, Head and Neck Surgery, University Hospital of Ioannina, Ioannina, GRC; 2 Speech and Language Therapy, University of Ioannina, Ioannina, GRC; 3 Otorhinolaryngology, Head and Neck Surgery, University Hospital of Alexandroupolis, Alexandroupolis, GRC

**Keywords:** volumetric trap, fungal spores, aerobiological studies, pollen calendar, airborne allergens

## Abstract

Introduction: In the region of Epirus (Northwestern Greece) there are characteristic climatic and geographical conditions that facilitate the growth of typical vegetation with the production of allergic pollen. Aerobiological research into airborne pollen diversity and seasonal variation in pollen counts and fungi spores has become essential due to the growing incidence of allergic rhinitis, allergic asthma, and other pollen-related and spore-related allergic conditions. Furthermore, weather conditions and other factors like air pollution may affect the intensity, the onset, and the duration of the pollen season, and the impact on the patient's symptomatology.

Methods: The diversity of airborne pollen grains (grains/m^3^) and fungal spores (spores/m^3^) in the region of Epirus were measured volumetrically using Burgard trap for 13 months, from May 1, 2017 to May 31, 2018.

Results: Totally, 10 pollen families and two fungi were recognized. The six most common taxa were Cupressaceae, Pinaceae, Urticaceae, Poaceae, Betulaceae, and Compositeae. The fungi taxa were Cladosporium and Alternaria. Peak pollen centralization was recorded from May to September. Urticaceae had the longest pollen season while Oleaceae and Pinaceae had the shorter. Fungal spores were recorded during all the months of the year. Also, there was a correlation between meteorological parameters and most pollen taxa.

Conclusion: The pollen and spore calendar shows the concentration of pollen grains and fungal spores in the region of Epirus. This knowledge is important for physicians and allergic patients as it could improve the management of the allergic respiratory disease.

## Introduction

Atmospheric pollen is a major cause of allergies, like allergic rhinitis, allergic asthma, and other allergic diseases which have become increasingly widespread in Europe over recent decades [1-3]. Airborne pollen grains are produced by anemogame plant species and they are significant organic aerosols. They, also, can be dispersed in the atmosphere and travel great distances [4-5]. It is a fact that airborne pollens are triggers of type I IgE-mediated allergic reaction in patients with allergic rhinitis and allergic bronchial asthma [5-7]. In each area, different species from the airborne pollen calendar which is dependent on the local geography, vegetation coverage, and meteorological conditions. Climatic factors, such as temperature, rainfall, humidity, and wind speed are modifiers of pollen dissemination [7-8]. As a result, the season of the different pollen types varies from country to country and from one region to another, depending on the climate and the vegetation [9].

Aerobiological studies are of great interest to physicians and allergy patients as a means of establishing a correlation between the aeroallergen concentration and allergic symptoms. This knowledge could help them to eliminate sources, adjust medication, and avoid exposure [5-6, 10]. Specific allergen proteins located on the pollen grain wall are responsible for immune system response and pollen-specific reaction [11]. Avoiding exposure to allergens constitutes the first and also the most important phase in the treatment of allergic diseases. Furthermore, allergic exacerbation caused by pollen exposure increases the socioeconomic burden of allergic disease.

Aerobiological studies have been developed rapidly in most parts of Europe and also in Greece. A correct and precise definition of the start and end of allergic pollen season is crucial for an adequate diagnostic approach [5, 12]. Pollen calendars are used by allergists to establish a cause-effect association between allergic sensitization to given pollen, demonstrated by IgE tests or skin prick tests and they have been constructed in many countries [12-14]. Nevertheless, there has not been recorded a pollen calendar for the region of Epirus until now. The research that is reported in this article aims to present the pollen and fungal spore dispersion in the atmosphere of the region of Epirus and to establish a preliminary pollen calendar for this area.

## Materials and methods

**Figure 1:**
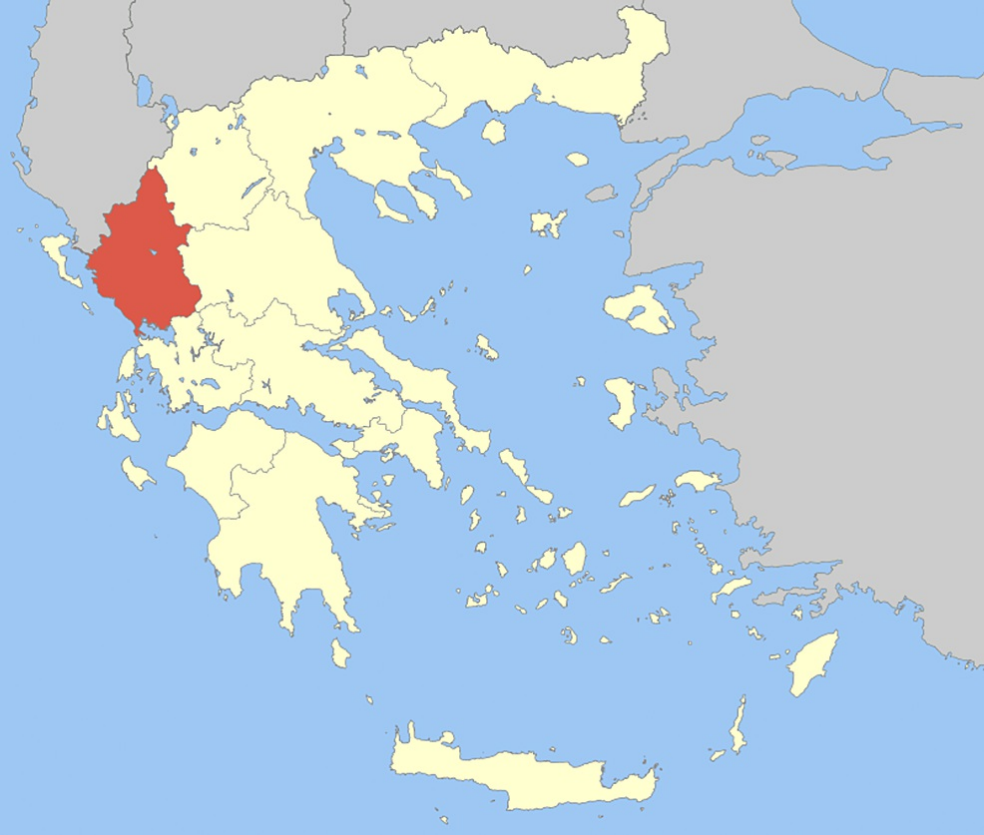
The region of Epirus (Greece) (el.wikipedia.org).

The Epirus region is located in Northwestern Greece; it has an area of about 9,200 km^2^ (3,600 sq mi) and it is largely made up of mountainous ridges. In the east, the Pindus Mountains that form the spine of mainland Greece, separate Epirus from Macedonia and Thessaly. Most of Epirus lies on the windward side of the Pindus. The winds from the Ionian Sea offer the region more rainfall than any other part of Greece. This area has a wide range of fauna and flora. The climate of Epirus is mainly Mediterranean and the vegetation is made up mostly of coniferous species (Figure 1).

In this study, airborne pollen grains and fungi spores were studied using a seven-day recording volumetric spore sampler (Burkard Scientific, Ltd., Middlesex, UK). The Burkard volumetric trap was placed 17 m above the ground, on the roof of the University Hospital of Ioannina (which is the capital of Epirus region) and about 500 m away from the nearest forest (two necessary conditions for an objective record of airborne allergens) [1-6]. The pollen trap was operating continuously for 13 months (from May 1, 2017 to May 31, 2018). A strip of silicone-coated Melinex tape was exposed to the air for trapping the pollen grains and fungi spores and was changed every 7 days. The tape was attached to a rotating drum which moved 2 mm/h completing a single revolution every one week. At the end of every 7-day cycle, the tape was removed and cut into 48-mm segments (one for a period of 24 h). These pieces were attached to glass slides using Gelvator to enable recognition of the aeroallergens under a high-resolution microscope at 400× magnification. Pollen grain and spore counts were expressed as pollen grains/m3 or spores/m3 of air and the pollen calendar was created according to the guidelines of the British Aerobiology Federation [1,13].

The meteorological data were provided by the station of the National Meteorological Service which is located in the area of Ioannina (Station number: 16642). Daily mean temperature (^0^C), humidity (%), rainfall (mm), and wind speed (km/h) were recorded. To be precise, the meteorological station is located at an altitude of 475 m above sea level and at the point with coordinates: latitude 39.69 and longitude 20.85. Regarding the measurement of the average daily rainfall, the data were not sufficient, as in about 40% of the recording days there were no data about rainfall. Therefore, because of the high risk of bias, only the three meteorological variables (temperature, humidity, and wind speed) were used. Then, meteorological parameters and pollen and fungi data were analyzed. Statistical analysis of data consists of two main axes. On the first axis, there is a descriptive analysis of the data. The appropriate tools of descriptive statistics were used to record the average value and the standard deviation of the concentrations of air allergens and meteorological conditions. In the second axis, multiple regressions were used to find out if there is a correlation between the three meteorological factors and the concentration of each type of aeroallergen. The IBM SPSS STATISTICS 22 (IBM, Armonk, NY) statistical package, a program widely used in statistical analysis, was used for data analysis. A value of p<0.05 was considered to be statistically significant.

## Results

During the 13-month aerobiological monitoring, a total of 10 different pollen types and two fungal spores were found. Among the pollen grains, six were arboreal taxa (Cupressaceae, Pinaceae, Betulaceae, Fagaceae, Oleaceae, and Platanaceae), four non-arboreal taxa (Urticaceae, Poaceae, Compositeae, and Chenopodiacea), and two fungal spores (Cladosporium and Alternaria). In Ioannina, the total pollen count (in 1 m3) was 9949.5 (pollen grains and fungal spores). Of the total pollen count in the study period, 2,257.2 were arboreal, 961.2 non-arboreal, and 6731,1 spores. The highest total (pollen grains and fungi spores) concentration of airborne allergens was recorded in August and the lowest in December. The period with the highest concentrations of airborne allergens (both pollen grains and fungal spores) was from May to September with a peak in August (Table 1).

**Table 1:** Total aeroallergens averages (total pollen grains/m3 and fungi spores/m3).

Month	Cupressaceae	Pinaceae	Fagaceae	Platanaceae	Betulaceae	Oleaceae	Poaceae	Compositeae	Chenopodiaceae	Urticaceae	Alternaria	Cladosporium	Total
May 17	30,78	100,98	43,2	17,82	36,18	18,9	67,5	0	0	69,12	78,84	96,12	559,44
June17	18,36	28,62	8,64	17,82	9,72	24,84	29,16	4,86	15,66	39,96	296,46	741,42	1235,52
July17	8,1	20,52	6,48	6,48	4,86	29,16	48,06	45,36	29,16	85,32	292,68	1037,88	1614,06
August 17	0	1,08	0	0	0	0,54	14,58	73,98	39,42	10,26	248,94	2158,38	2547,18
September 17	0	7,02	2,7	0	0	0	0	1,62	3,24	3,24	71,82	273,78	363,42
October 17	0	16,2	0	0	0	0	0	7,02	16,2	14,04	52,92	220,86	327,24
November17	0,54	14,04	17,82	2,7	1,08	5,4	9,72	23,22	20,52	31,86	77,76	178,2	382,86
December 17	7,02	4,86	3,78	0	0	0	0	9,18	15,66	27,54	23,22	172,26	263,52
January 18	216,54	0	0	0	0,54	0	0,54	2,7	2,7	3,24	26,46	81,54	334,26
February18	304,02	0	2,7	0	63,72	0	2,16	0	0,54	0	55,62	65,88	494,64
March 18	265,68	0	0	7,02	97,2	0	65,34	0	0	0	68,58	62,1	565,92
April 18	196,56	207,36	17,28	19,98	12,96	1,08	20,52	0	2,7	3,78	38,88	52,38	573,48
May 18	88,56	125,82	31,32	22,68	41,04	18,9	58,32	0	0	43,2	103,68	154,44	687,96
Total	1136,16	526,5	133,92	94,5	267,3	98,82	315,9	167,94	145,8	331,56	1435,86	5295,24	9949,5
Means	87,4	40,5	10,3	7,27	20,56	7,6	24,3	12,92	11,22	25,5	110,45	407,33	1421,36
%	11,42	5,29	1,35	0,95	2,69	0,99	3,18	1,69	1,47	3,33	14,43	53,22	100

**Figure 2:**
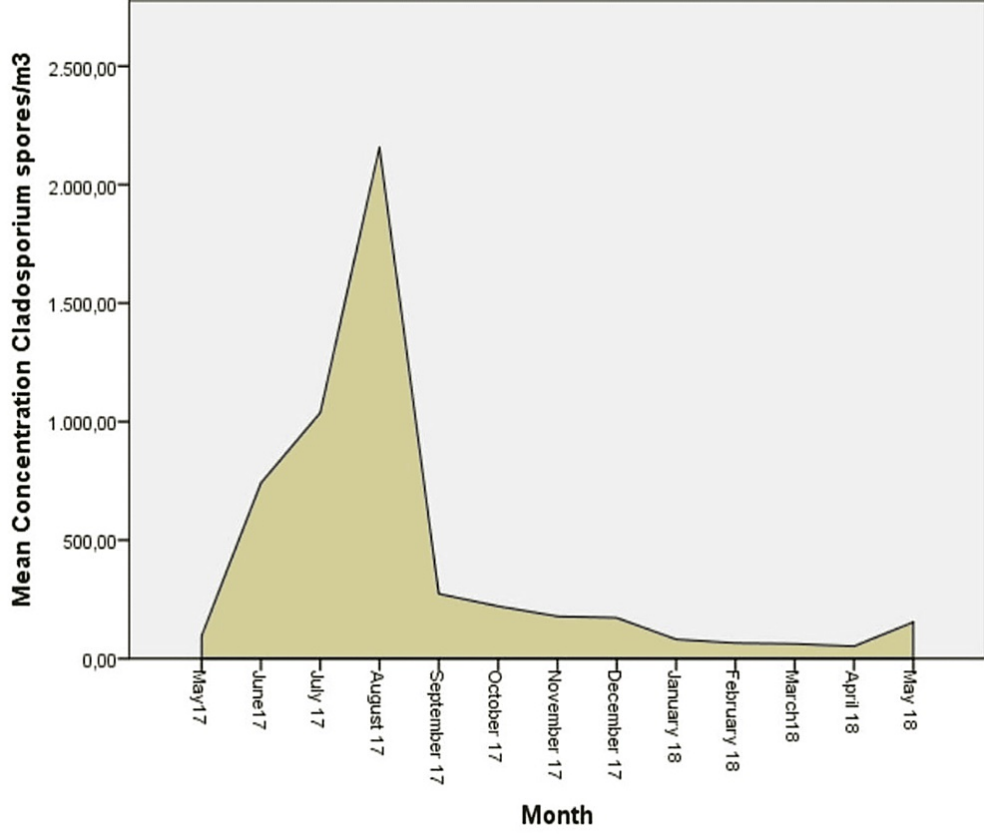
Daily mean concentrations for Cladosporium (spores/m3), the most abundant aeroallergen.

**Figure 3:**
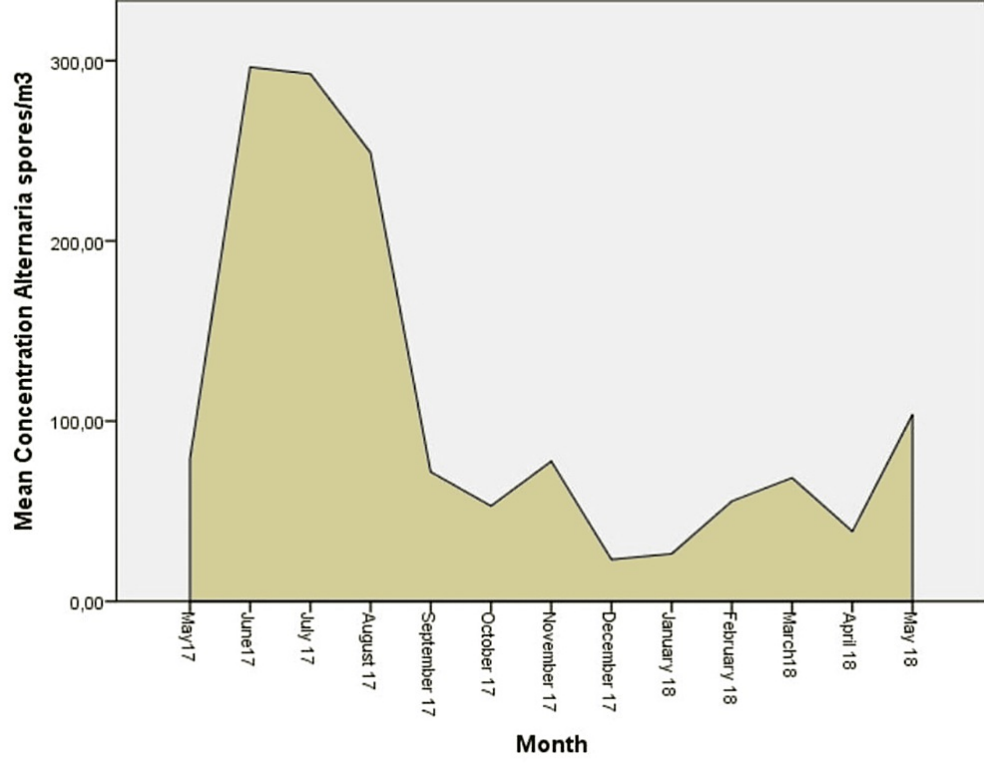
Daily mean concentrations for Alternaria (spores/m3), the second more abundant aeroallergen.

**Figure 4:**
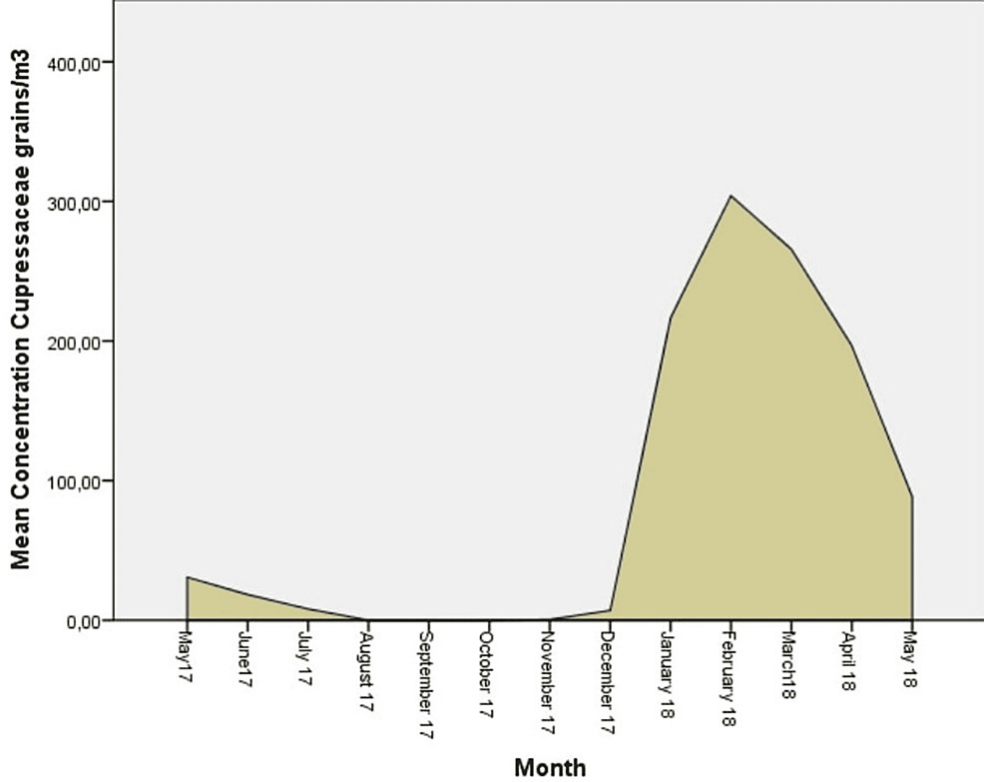
Daily mean concentrations for Cupressaceae (grains/m3), the third more abundant aeroallergen.

**Figure 5:**
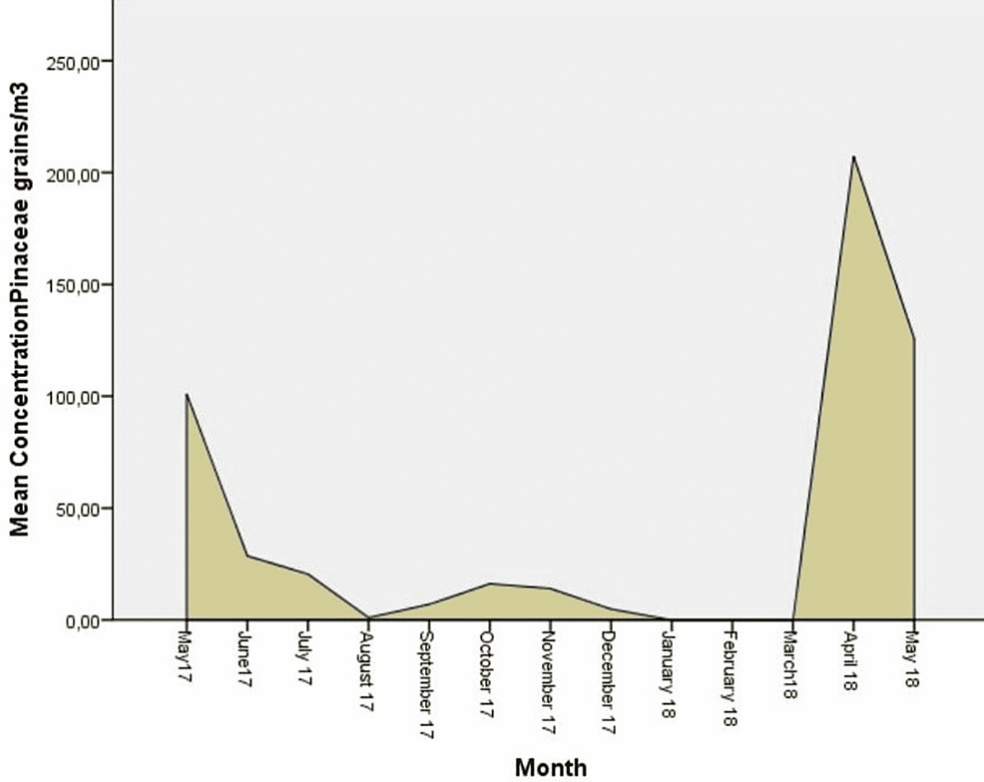
Daily mean concentrations for Pinaceae (grains/m3), the fourth more abundant aeroallergen.

**Figure 6:**
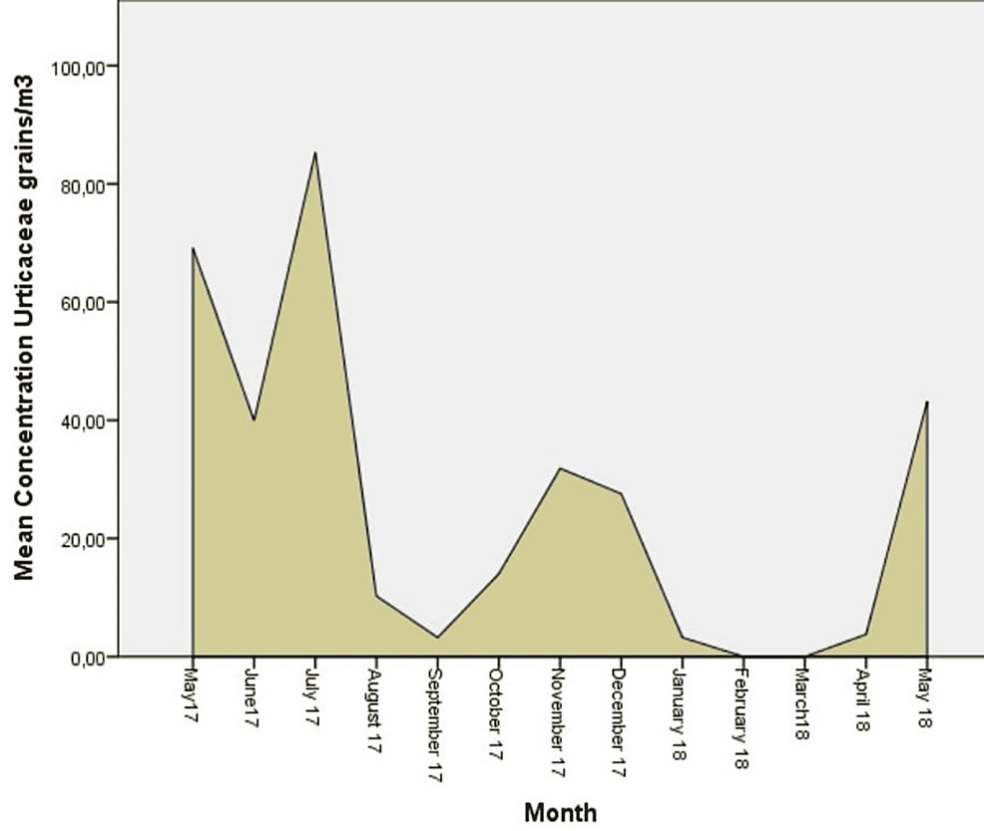
Daily mean concentrations for Urticaceae (grains/m3), the fifth more abundant aeroallergen.

**Figure 7:**
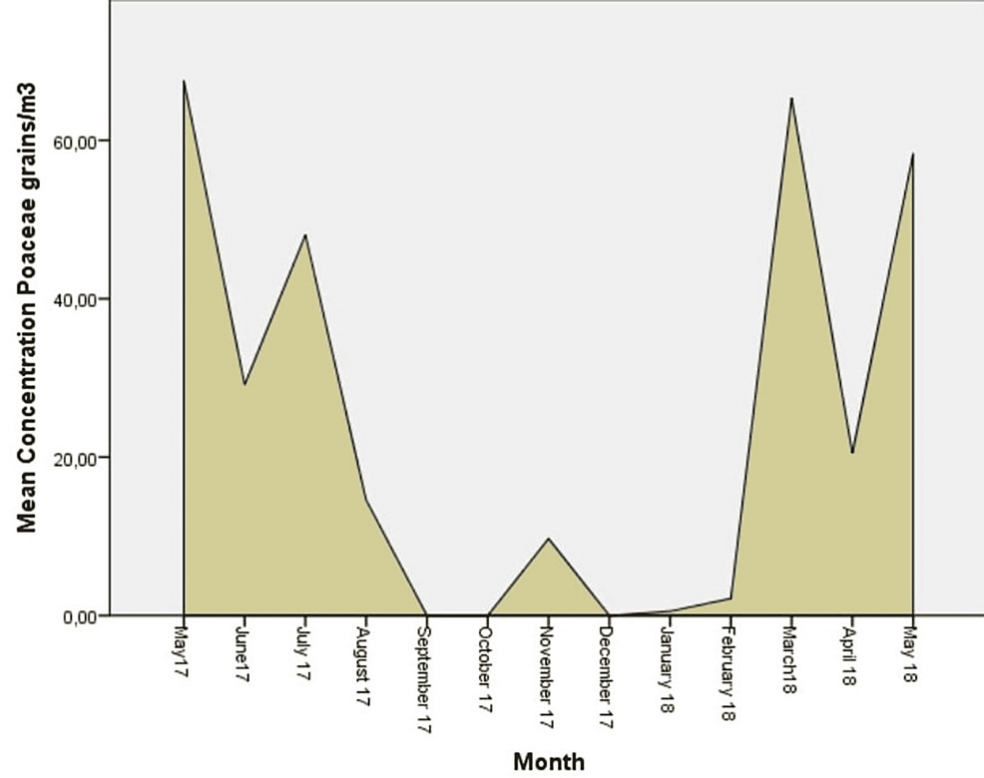
Daily mean concentrations for Poaceae (grains/m3), the sixth more abundant aeroallergen.

The predominant aeroallergens in the airborne spectrum were fungi spores Cladosporium (53.22%) and Alternaria (14.43%). Regarding the pollen grains, the highest average total concentrations were recorded for Cupressaceae (11.42%), Pinaceae (5.29%), Urticaceae (3.33%), Poacea (3.18%), and Betulaceae (2.69%). Pollen grains were recorded at lower concentrations for Composiateae (1.69%), Chenopodiaceae (1.47%), Fagaceae (1.35%), Oleacea (0.99%), and Platanaceae (0.95%) (Figures 2-7).

Moreover, fungal spores were recorded all the months of the year. The highest concentration of Cladosporium and Alternaria was recorded in summer, with its peak in August for Cladosporium (2,158.38 grains/m3) and in June for Alternaria (296.46 grains/m3). The main spore season for fungi in the atmosphere of the region of Epirus was observed from May to November. 

**Figure 8:**
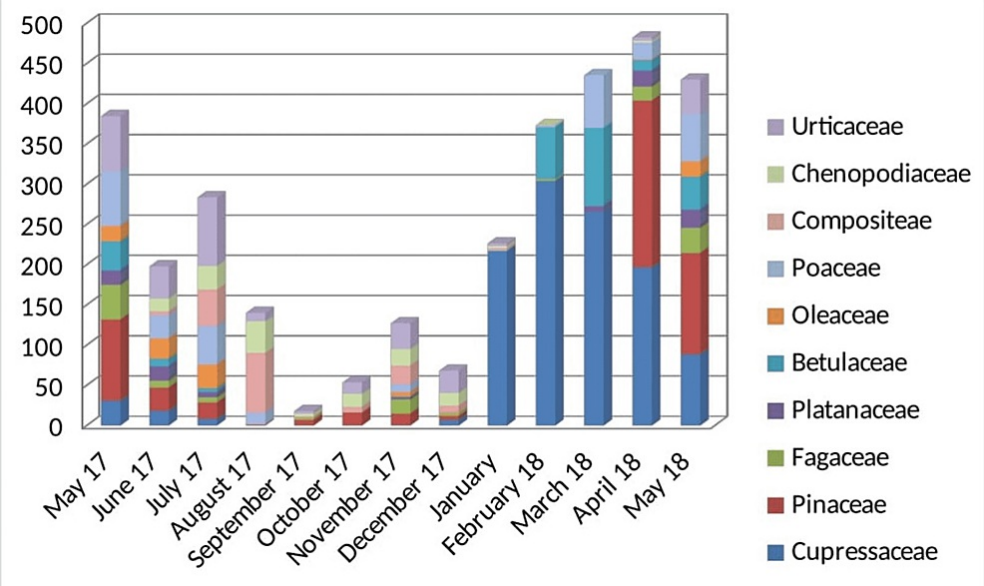
The concentration of pollen grains (grains/m3) and the pollen seasons.

Concerning the counts of pollen grains, two peaks were found throughout the study period. The highest concentration was found from January to May with a peak in April (482.22 grains/m3) and minimum levels in September (17.82 grains/m3). During this period (from January to May) there is a predominance of pollen grains of Cupressaceae and Pinaceae. In July, there is a second peak due to the high levels of Urticaceae (85.32 grains/m3), Poaceae (48.06 grains/m3), and Compositeae (45.36 grains/m3) (Figure 8).

In the present study, three meteorological factors, namely average daily temperature (^o^C), relative humidity (%), and wind speed (knot) were analyzed. Based on the data, July was the warmest (27.52 ± 2.29^o^C) month and August the driest (41.28 ± 7.70%). On the other hand, November was the coldest (5.11 ± 3.40^o^C) month and January the wettest (84.85%). Also, February (4.00 ± 2.46 knot) was the windiest month and May was the calmest (0.78 ± 1.21) (Table 2).

**Table 2:** Climatic conditions (Means ± SD). SD, standard deviation

	May 17	June 17	July 17	August 17	September 17	October 17	November 17	December 17	January 18	February 18	March 18	April 18	May 18	Means (SD)
Temperature (^ο^C)	24.10 (2.86)	27.22 (2.31)	27.52 (2.29)	19.84 (2.54)	14.82 (2.35)	9.53 (2.68)	5.11 (3.40)	5.57 (1.86)	7.18 (2.04)	9.66 (2.70)	17.07 (2.92)	19.00 (2.00)	24.10 (2.47)	16.21 (8.13)
Humidity (%)	77.61 (9.58)	58.20 (7.48)	45.42 (5.65)	41.28 (7.70)	60.00 (9.38)	66.41 (10.50)	81.03 (15.66)	81.19 (8.17)	84.85 (11.38)	82.72 (12.75)	78.44 (8.66)	63.37 (9.45)	77.61 (8.66)	69.09 (17.15)
Wind speed (knots)	0.78 (1.21)	2.52 (1.08)	2.95 (1.09)	2.25 (1.47)	2.23 (0.98)	1.52 (1.02)	1.19 (2.71)	2.77 (2.97)	3 (2.80)	4.00 (2.46)	3.68 (1.87)	2.43 (0.96)	0.79 (0.69)	2.32 (2.01)

The correlation between the three meteorological parameters (temperature, humidity, wind speed) and the pollen concentration was significant in a large number of cases (Table 3). The data analysis revealed a statistically significant positive correlation for Fagaceae (p=0.007<0.05), Platanaceae (p=0.012<0.05), Betulaceae (p=0.031<0.05), Oleaceae (p=0.008<0.05), and Poaceae (p=0.007<0.05) with mean daily temperature. Furthermore, Platanaceae (p=0.018<0.05), Fagaceae (p=0.004<0.05), Oleacea (p=0.032<0.05), and Poaceae (p=0.012<0.05) were associated positively with relative humidity. Equally, Cupressaceae (p=0.008<0.05) had a statistically significant positive correlation with wind speed. On the contrary, the correlation of Compositeae (p=0.049<0.05) and Chenopodiaceae (p=0.011<0.05) with relative humidity was negative. The data analysis found no statistically significant correlation with any of the meteorological parameters for Pinaceae, Urticaceae, Alternaria, and Cladosporium.

**Table 3:** Impact of climatic factors on atmospheric concentration of pollen and spores. (Results from statistical analysis. A value of p<0.05 was considered to be statistically significant).

	Beta			t			p value		
Pollen/Spores	Temperature (^0^C)	Humidity (%)	Wind speed (knots)	Temperature (^0^C)	Humidity (%)	Wind speed (knots)	Temperature (^0^C)	Humidity (%)	Wind speed (knots)
Cupressaceae	0.700	1.102	0.836	1.343	2.216	3.419	0.212	0.054	0.008
Pinaceae	0.742	0.658	-0.199	0.923	0.858	-0.529	0.380	0.413	0.610
Fagaceae	1.367	1.442	-0.309	3.441	3.803	-1.659	0.007	0.004	0.131
Platanaceae	1.835	1.609	0.138	3.154	2.898	0.505	0.012	0.018	0.626
Betulaceae	1.532	1.760	0.804	2.557	3.078	2.862	0.031	0.013	0.019
Oleaceae	1.875	1.347	0.265	3.362	2.531	1.015	0.008	0.032	0.337
Poaceae	2.046	1.778	0.393	3.447	3.139	1.413	0.007	0.012	0.191
Compositeae	-0.692	-1.323	-0.215	-1.133	-2.269	-0.751	0.287	0.049	0.472
Chenopodiaceae	-1.069	-1.669	-0.379	-1.985	-3.166	-1.462	0.078	0.011	0.178
Urticaceae	1.164	0.799	-0.072	1.628	1.170	-0.214	0.138	0.272	0.835
Alternaria	1.033	0.133	0.337	2.215	0.300	1.541	0.054	0.771	0.158
Cladosporium	-0.137	-0.948	-0.015	-0.264	-1.913	-0.061	0.798	0.088	0.953
Total	0.465	-0.406	0.198	0.914	-0.836	0.831	0.384	0.425	0.428

## Discussion

Allergic respiratory diseases (allergic rhinitis and allergic asthma) are major public health problems, with high prevalence in the general population varying significantly among the countries from 18.1% to 39.0%. They are IgE-mediated inflammatory chronic disorders caused by exposure to airborne allergens with an increasing trend over the last years [14-17]. The quality of life is considered to be negatively affected by allergic respiratory diseases [18-19]. Also, allergic rhinitis and allergic asthma represent a substantial burden of morbidity and health service costs. In addition to the direct medical costs of allergic respiratory disease, there are other indirect costs that include days off from work and poor productivity. The management of respiratory allergy is initially, based on allergen avoidance (when possible). Patients should be educated about their condition and be advised to avoid known aeroallergens [20-21].

Pollen grains and fungi spores have detrimental effects on health and it is logical that these airborne allergenic particles are monitored throughout the world. The data of this monitoring must be available to the public. In the region of Epirus, there are characteristic climatic conditions that facilitate the growth of typical vegetation and the production of pollen allergens. The pollen grains registered in the atmosphere of Ioannina (the capital of the region of Epirus) come from the plants growing in this area as well as areas several kilometers distant. The aerobiologic sampling of the pollen content of the air in Ioannina, carried out from May 1, 2017 to May 31, 2018, and led us to identify the most common aeroallergens in the atmosphere of this area. The results of the recording of airborne pollen and fungal spores showed that the highest concentration (grains/m3 or spores/m3) had the fungal spores Cladosporium, which had an average total concentration of 407.33 spores/m3 (53.22% of all airborne allergens) and Alternaria with 110.45 spores/m3 (14.43% of all air allergens). Regarding the pollen grains, the highest average total concentration was recorded for the species Cupressaceae with 87.4 (11.42%), Pinaceae with 40.5 (5.29%), Urticaceae with 25.5 (3.33%), Poacea with 24.3 (3.18%), and Betulaceae with 20.5 (2.69%). Pollen grains were recorded at lower concentrations for Composiateae with 12.92 (1.69%), Chenopodiaceae with 11.22 (1.47%), Fagaceae with 10.3 (1.35%), Oleacea with 7.6 (0.99%), and Platanaceae with 7.27 (0.95%). Additionally, the period with the highest total concentration of airborne particles was recorded from May to September. Fungal spores were recorded all the year but for the pollen grains, there were two peaks through the study period. The first peak was found in April with a predominance of pollen grains of Cupressaceae and Pinaceae and the second peak in July due to the high levels of Urticaceae, Poaceae, and Compositeae. Concerning to the meteorological parameters (average daily temperature, relative humidity, and wind speed), the data analysis revealed a statistically significant (p<0.05) correlation for Fagacea, Platanaceae, Betulaceae, Oleacea, Cupressaceae, Compositeae, and Chenopodiaceae. On contrary, the data analysis found no statistically significant correlation with any of the meteorological parameters for Pinaceae, Urticaceae, Alternaria, and Cladosporium.

In the region of Epirus, no data about the pollen calendar or the relation between pollen concentrations and meteorology were published prior to this study. The knowledge of the pollen and spore calendar of this region is important for allergic patients and it is the most reliable way to assess the health hazard for them. Also, it is crucial that patients and physicians can take necessary precautions (avoid exposures, medications). The avoidance strategies could reduce allergen exposure and prevent the elicitation of allergic symptoms.

## Conclusions

Pollen grain and fungi spore forecasting is an active research ground. Knowledge of pollen and spore calendar may prove useful for physicians and allergic patients and management (avoidance and medical treatment) of allergic respiratory disease. Minimizing exposure to airborne allergens might reduce allergic exacerbations of allergic rhinitis and allergic asthma. The present aerobiological study is the first in the region of Epirus. Further aerobiological research has to be conducted in the future, in order to confirm the conclusions of this study and to create the pollen and spore calendars for all the regions of Greece. Additionally, as the aeroallergens concentration is closely related to meteorological parameters, which vary throughout the years, a comparative analysis of data from several consecutive years is suggested to be done.

## References

[1] Herminia Garcia-Mozo, Rosa Pèrez-Badia, Federico Fernández-González, Carman Galán (2006). Airborne pollen sampling in Toledo, Central Spain. Aerobiologia.

[2] Beggs PJ (2004). Impacts of climate change on aeroallergens: past and future. Clin Exp Allergy.

[3] Frenz DA (2001). Interpreting atmospheric pollen counts for use in clinical allergy: allergic symptomology.. Ann Allergy Asthma Immunol.

[4] Bousquet J, Anto J, Auffray C (2011). MeDALL (Mechanisms of the Development of ALLergy): an integrated approach from phenotypes to systems medicine. Allergy.

[5] D'Amato G, Cecchi L, Bonini S (2007). Allergenic pollen and pollen allergy in Europe. Allergy.

[6] Al-Frayh AR, Reilly H, Harfi HA (1989). A 12-month aerobiological survey of pollen in Riyadh. Annals of Saudi Mediane..

[7] Arshad SH, Karmaus W, Matthews S (2001). Association of allergy-related symptoms with sensitisation to common allergens in an adult European population. J Investig Allergol Clin Immunol.

[8] Baran H, Ozcan KM, Selcuk A, Cetin MA, Cayir S, Ozcan M, Dere H (2014). Allergic rhinitis and its impact on asthma classification correlations. J Laryngol Otol.

[9] D’Amato G., Lobefalo G (1989). Allergenic pollen in the southern Mediterranean. J Allergy Clin Immunol.

[10] Türe C, Böcük H (2009). Analysis of airborne pollen grains in Bilecik, Turkey. Environ Monit Assess.

[11] Hauser M, Roulias A, Ferreira F, Egger M (2010). Panallergens and their impact on the allergic patient. Allergy Asthma Clin Immunol.

[12] Skoner DP (2001). Allergic rhinitis: definition, epidemiology, pathophysiology, detection, and diagnosis. J Allergy Clin Immunol.

[13] Katotomichelakis M, Nikolaidis C, Makris M (2015). The clinical significance of the pollen calendar of the Western Thrace/northeast Greece region in allergic rhinitis. Int Forum Allergy Rhinol.

[14] Geller-Bernstein C, Portnoy JM (2019). The clinical utility of pollen counts. Clin Rev Allergy Immunol.

[15] Ebo DG, Hagendorens MM, De Knop KJ, Verweij MM, Bridts CH, De Clerck LS, Stevens WJ (2010). Component-resolved diagnosis from latex allergy by microarray. Clin Exp Allergy.

[16] Dykewicz MS, Hamilos DL (2010). Rhinitis and sinusitis. J Allergy Clin Immunol.

[17] Bousquet J, Khaltaev N, Cruz AA (2008). Allergic rhinitis and its impact on asthma (ARIA) 2008 update (in collaboration with the World Health Organization, GA(2)LEN and AllerGen). Allergy.

[18] Krouse HJ, Davis JE, Krouse JH (2002). Immune mediators in allergic rhinitis and sleep. Otolaryngol Head Neck Surg.

[19] Coban H, Aydemir Y (2014). The relationship between allergy and asthma control, quality of life, and emotional status in patients with asthma: a cross-sectional study. Allergy Asthma Clin Immunol.

[20] Dahl R, Andersen PS, Chivato T, Valovirta E, de Monchy J (2004). National prevalence of respiratory allergic disorders. Respir Med.

[21] Fleming DM, Crombie DL (1987). Prevalence of asthma and hay fever in England and Wales. Br Med J (Clin Res Ed).

